# Clinical Characteristics and Efficacy of Adalimumab and Low-Dose Methotrexate Combination Therapy in Patients With Vogt–Koyanagi–Harada Disease

**DOI:** 10.3389/fmed.2021.730215

**Published:** 2022-01-06

**Authors:** Tomona Hiyama, Yosuke Harada, Yoshiaki Kiuchi

**Affiliations:** Department of Ophthalmology and Visual Science, Graduate School of Biomedical Sciences, Hiroshima University, Hiroshima, Japan

**Keywords:** Vogt-Koyanagi-Harada disease, uveitis, adalimumab, methotrexate, cyclosporine A, tumor necrosis factor-alpha

## Abstract

This retrospective study investigated the clinical characteristics and efficacy of adalimumab and low-dose methotrexate combination therapy in patients with Vogt–Koyanagi–Harada disease who were treated at Hiroshima University from February 2012 to May 2021. The patients' demographics, clinical features at administration of immunosuppressive therapy, steroid-sparing immunosuppressive therapy, side effects, and relapses were recorded. The efficacies of steroid-sparing immunosuppressive therapy (methotrexate, cyclosporine A, adalimumab, and adalimumab and methotrexate combination therapy) were analyzed. Among 62 patients, the median age at diagnosis was 47 years and the median duration of uveitis was 51 months. Systemic corticosteroid therapy was administered to 93.5% of patients (*n* = 58). Thirty-four patients (54.8%) were treated with steroid-sparing immunosuppressive therapy. Methotrexate and cyclosporine A were administered to 12 and 22 patients, respectively; relapse occurred in 50.0% and 22.7% of the patients, respectively. Discontinuation of cyclosporine A was required in 63.6% of patients because of side effects. Adalimumab was administered to 14 patients. Recurrence occurred in 11 patients, requiring methotrexate concomitantly. The mean dose of methotrexate at inflammatory quiescence after side effect-related dose decrease was 8.0 mg/week (0.13 mg/kg). The median duration of combination therapy without recurrence was 20 months. There were no serious adverse events during adalimumab therapy. A high relapse rate was observed in patients receiving methotrexate; a high rate of side effects requiring discontinuation was observed in patients receiving Cyclosporine A. Patients with late-stage Vogt–Koyanagi–Harada disease may achieve better control with adalimumab and methotrexate combination therapy.

## Introduction

Vogt–Koyanagi–Harada disease (VKH) is an autoimmune bilateral panuveitis often associated with neurological and cutaneous involvement (1). In the early-stage of disease, granulomatous choroiditis occurs with exudative retinal detachment and optic disc swelling, causing a bilateral reduction of visual acuity (2, 3). Late-stage disease occurs in patients with insufficient treatment; this phase is characterized by depigmentation signs such as “sunset glow fundus” and recurrent uveitis, with an increased risk of ocular complications (1–4). Early and aggressive systemic corticosteroid therapy is the basic treatment; however, systemic corticosteroid therapy alone cannot prevent the onset of late-stage disease (1, 5). The early administration of additional steroid-sparing immunosuppressive therapy within 2–3 weeks of onset was reported to prevent the onset of late-stage VKH (6). Recently, mycophenolate mofetil and mycophenolic acid were found to prevent the onset of chronic VKH (7). Unfortunately, the choice of immunosuppressive therapy depends on drug availability and local regulations—in Japan, only cyclosporine A (CsA) and adalimumab (ADA) are approved for the treatment of non-infectious uveitis. VKH is the second most diagnosed intraocular inflammatory disease after sarcoidosis in Japan; many affected patients are treated solely with systemic corticosteroids at general ophthalmology hospitals. Although vigorous early immunosuppressive therapy is recommended to prevent the onset of late-stage VKH, it is not always possible in clinical practice because of treatment availability and cost. Furthermore, it is challenging to control ocular inflammation in patients with late-stage VKH (8). In this study, we analyzed the clinical characteristics and treatment efficacies of immunosuppressive therapies in patients with VKH and focused on the treatment of patients with late-stage VKH.

## Materials and Methods

This retrospective study reviewed data from patients with VKH who were treated at Hiroshima University Hospital (Hiroshima, Japan) from February 2012 to May 2021. The study was performed with approval from the University of Hiroshima Institutional Review Board. Written informed consent was obtained from all patients. Permission was granted by the Evaluation Committee on Unapproved or Off-labeled Drugs with High Medical Needs at Hiroshima University for methotrexate (MTX) for patients with non-infectious uveitis.

VKH was diagnosed in accordance with international diagnostic criteria (9). Patients were initially treated with oral corticosteroids or intravenous pulse corticosteroid therapy (1,000 mg methylprednisolone per day for 3 consecutive days) followed by the tapering of oral corticosteroids. Topical treatments were used if necessary. The study included all patients who were followed up for more than 6 months. Patients were excluded if they had an unknown date of onset or were lost to follow-up during treatment. Early-stage and late-stage VKH were diagnosed according to the classification criteria for VKH by the standardization of uveitis nomenclature working group (3).

The data collected in this study included patient age at diagnosis, sex, duration of uveitis, initial treatment, use of steroid-sparing immunosuppressive therapy, time until the first immunosuppressive therapy, dose of oral corticosteroids at baseline, ocular findings at baseline (anterior chamber cells ≥1+, posterior synechiae, exudative retinal detachment, choroidal thickening/folding, sunset glow fundus, chorioretinal atrophy, choroidal neovascularization), neurological (headache/tinnitus/dysacusis/meningismus/cerebrospinal fluid pleocytosis)/cutaneous (vitiligo, poliosis, alopecia) features at baseline, best-corrected visual acuity at baseline, relapse during the immunosuppressive therapy, and side effects of immunosuppressive therapy. Inflammatory relapse was defined as the presence of the following: anterior chamber cells ≥1+ (according to Standardization of Uveitis Nomenclature Working Group criteria), increase in subfoveal choroidal thickness or choroidal folding based on enhanced depth imaging optical coherence tomography, and indocyanine green angiography signs (e.g., choroidal vasculitis and hypofluorescent dark dots representing choroidal granulomas) (10). For patients treated with ADA and MTX combination therapy, data were obtained including previous immunosuppressive therapy, duration from onset of VKH to ADA administration, duration from ADA administration to MTX administration, dose of concomitant MTX, relapse after the combination therapy, and relapse-free period under the combination therapy.

Prior to the administration of immunosuppressive therapies (e.g., MTX, CsA, and ADA), an extensive work-up was performed including complete blood count, hepatic and renal function tests, exclusion of tuberculosis and syphilis, hepatitis B and C serology, serum angiotensin converting enzyme, and chest radiography. Laboratory tests and chest radiography were performed regularly during immunosuppressive therapy. CsA blood concentration was monitored regularly. Electrocardiograms were routinely performed before starting ADA for screening heart disease (11, 12). An initial dose of 80 mg ADA was administered subcutaneously, followed by 40 mg ADA subcutaneously at 2-week intervals, beginning 1 week after the initial dose. All patients were examined by several ophthalmologists at each visit at intervals of ≤2 months. ADA was initiated when conventional therapy (i.e., oral corticosteroids >5 mg, with or without oral immunosuppressants) failed to control ocular inflammation or when patients could not tolerate corticosteroids (e.g., in patients with diabetes mellitus or steroid-induced glaucoma) or other oral immunosuppressants (MTX or CsA). MTX or CsA were discontinued at administration of ADA. On the basis of factors such as age, severity of ocular inflammation, and range of ocular complications, some patients received ADA as a first-line steroid-sparing immunosuppressive therapy. Concomitant MTX was administered when inflammatory relapse occurred in patients who were treated with ADA.

Statistical analysis was performed using Microsoft Excel (Microsoft Corporation, Redmond, WA, USA) and JMP Pro, version 15 (SAS, Cary, NC, USA). Quantitative variables were summarized using counts (percentages), as well as means and medians (ranges). Qualitative variables were analyzed using Fisher's exact test. Differences with *P*-values < 0.05 were considered statistically significant.

## Results

Overall, 62 patients (25 men, 37 women) with VKH were included in the study. The patient demographics are summarized in Table 1. The median age at diagnosis was 47 years (range, 18–77 years). The duration of uveitis was 51 months (range, 15–148 months). Systemic corticosteroid therapy was administered in 93.5% of the patients (*n* = 58). Thirty-four patients (54.8 %) were treated with steroid-sparing immunosuppressive therapy, 10 were administered treatment at the early-stage of the disease, and 24 were administered treatment at the late-stage of disease.

**Table 1:** Demographics of patients with Vogt–Koyanagi–Harada disease.

**Characteristics**	***n***	**%**
Patients	62	
Male	25	36.8
Female	37	54.4
Age at diagnosis, median in years (range)	47 (18–77)	
Duration of uveitis, median in months (range)	51 (15–148)	
Initial treatment of Vogt–Koyanagi–Harada disease onset		
Pulse corticosteroid therapy	58	93.5
Oral corticosteroid thearpy	4	6.5

The baseline clinical characteristics of patients with early-stage (*n* = 10) and late-stage (*n* = 24) VKH at immunosuppressive therapy administration are summarized in Table 2. The median dose of oral corticosteroids at baseline was 37.5 mg and 11.3 mg in early- and late-stage VKH, respectively. The most common first choice immunosuppressive therapy was CsA in both groups. Anterior chamber cells >1+ were present in more than 50% of patients in both groups. Exudative retinal detachment was present in 80.0% of the patients with early-stage VKH. At baseline, choroidal thickening/folding were observed in ~80% of the patients in the early- and late-stage VKH groups. All patients with late-stage VKH presented with sunset glow fundus. Best-corrected visual acuity was better than 20/50 in more than 80% of the eyes in both groups.

**Table 2:** Baseline clinical characteristics of patients with Early-Stage and Late-Stage Vogt-Koyanagi-Harada disease at time of administration of immunosuppressive therapy.

	**Early-stage**	**Late-stage**
**Clinical characteristics**	***n*** **= 10**	***n*** **= 24**
Duration from onset to first immunosuppressive therapy, Median in months (range)	0.5 (0–2)	8 (0–105)
Dose of oral corticosteroids at baseline, Median in milligram (range)	37.5 (20–50)	11.3 (0–60)
First choice immunosuppressive therapy		
Methotrexate	1	4
Cyclosporine A	8	13
Adalimumab	1	7
Ocular findings at baseline		
Anterior chamber cell (%)	7 (70.0)	14 (58.3)
Posterior synechiae (%)	1 (10.0)	2 (8.3)
Exudative retinal detachment (%)	8 (80.0)	4 (16.7)
Choroidal thickening/folding (%)	8 (80.0)	19 (79.2)
Sunset glow fundus (%)	0 (0)	24 (100)
Chorioretinal atrophy (%)	0 (0)	6 (25.0)
Choroidal neovascularization (%)	0 (0)	2 (8.3)
Neurological^*a*^/Cutaneous^*b*^ features at baseline (%)	2 (20.0)	7 (29.2)
Best corrected visual acuity at baseline	20 eyes	48 eyes
Better than 20/50 (%)	16 (80.0)	44 (91.7)
20/50 to 20/200 (%)	3 (15.0)	3 (6.3)
Worse than 20/200 (%)	1 (5.0)	1 (2.1)

a *Headache/tinnitus/dysacusis/meningismus/cerebrospinal fluid pleocytosis*.

b *Vitiligo/poliosis/alopecia*.

Table 3 shows the details of immunosuppressive therapy administered to patients with VKH disease. MTX was administered to 12 patients; 11 of whom were administered MTX as a second choice. Relapse during MTX treatment occurred in 50% (*n* = 6) of the patients. MTX was discontinued in 25.0% (*n* = 3) of the patients because of side effects (alopecia, myelosuppression, nephrotoxicity). Of 22 patients who received CsA, 21 were administered it as a first choice. Relapse occurred in 22.7% (*n* = 5) of the patients. The discontinuation of CsA because of side effects occurred in 63.6% (*n* = 14) of the patients (nephrotoxicity, myelosuppression, hepatotoxicity, hematuria, edema, numbness, fever); this rate of side effects was higher than that among patients who received MTX (Fisher's exact test, *P* = 0.0354). The median dose of CsA at which side effects occurred was 137.5 mg/day (2.3 mg/kg). ADA was administered to 14 patients; as the first choice in 8 patients, as the second choice in 4 patients, and as the third choice in 2 patients. Of 14 patients, 13 were administered ADA at late-stage disease. Relapse during ADA occurred in 11 patients, who then required concomitant MTX. No side effects were observed during ADA therapy.

**Table 3:** Immunosuppressive therapy in patients with Vogt–Koyanagi–Harada disease.

	***n***	**%**
**Methotrexate (*****n*** **=** **12)**		
First choice	1	8.3
Second choice	11	91.7
Early-stage	5	41.7
Late-stage	7	58.3
Relapse during methotrexate therapy	6	50.0
Discontinuation due to side effects	3	25.0
**Cyclosporine A (*****n*** **=** **22)**		
First choice	21	95.5
Second choice	1	4.5
Early-stage	8	36.4
Late-stage	14	63.6
Relapse during cyclosporine A therapy	5	22.7
Discontinuation due to side effects	14	63.6
**Adalimumab (*****n*** **=** **14)**		
First choice	8	57.1
Second choice	4	28.6
Third choice	2	14.3
Early-stage	1	7.1
Late-stage	13	92.9
Relapse during Adalimumab therapy	11	78.6
Discontinuation due to side effects	0	0.0
Monotherapy	3	21.4
Adalimumab and Methotrexate combination therapy	11	78.6

The 11 patients treated with ADA and MTX combination therapy following relapse during ADA monotherapy are summarized in Table 4. All 11 patients were administered ADA at late-stage disease. The median duration from onset to ADA administration was 5 months (range, 0–105 months). Nine out of 11 patients relapsed within the first 6 months after starting ADA, and received concomitant MTX. All 11 patients had received MTX by 12 months (median, 2 months; range, 0–12 months). The mean maximum dose of concomitant MTX was 11.5 mg/week (0.18 mg/kg). A dose adjustment of MTX was required in five patients (45.5%) because of side effects (hepatotoxicity and fatigue). The mean dose of MTX at inflammatory quiescence after dose adjustment was 8.0 mg/week (0.13 mg/kg). Inflammatory relapse occurred in three patients (27.3%) after 4, 6, and 12 months of combination therapy (patients 6, 10, and 11): subsequently, the MTX dose was increased in these three patients. The median duration of ADA and MTX combination therapy without relapse was 20 months (range, 4–31).

**Table 4:** Patients treated with adalimumab and methotrexate combination therapy.

**Patient**	**Sex**	**Age at diagnosis (years)**	**Duration of uveitis (months)**	**Previous immuno** **suppressive therapy**	**Duration from onset to ADA administration (months)**	**Duration from ADA to MTX administration (months)**	**MTX dose at inflammatory quiescence (mg)**	**Side effects of MTX that required dose adjustment**	**Relapse after combination therapy**	**Combination therapy without relapse (months)**
1	F	59	19	+	5	2	10		–	12
2	F	56	56	–	14	12	4	Hepatotoxicity	–	30
3	F	45	110	+	85	0	8	Fatigue	–	25
4	M	38	24	–	0	3	8		–	20
5	F	29	141	+	105	7	8		–	28
6	M	50	57	+	23	2	14		+	12
7	M	37	36	–	2	2	4	Hepatotoxicity	–	31
8	M	42	37	–	3	2	4	Hepatotoxicity	–	31
9	M	27	20	+	8	0	4	Hepatotoxicity	–	16
10	M	22	12	–	2	0	16		+	6
11	M	59	11	–	0	0	16		+	4

*M, male; F, female; ADA, adalimumab; MTX, methotrexate*.

There were no serious adverse events associated with ADA treatment in this study. Side effects occurred in 11 out of 21 patients (52.4%) who received MTX (including MTX monotherapy and combination therapy). Side effects were managed by dose adjustment in 8 out of 21 patients (38.1%), and, 14.3% (*n* = 3) discontinued MTX. In contrast, side effects of CsA that required discontinuation were observed in 63.6% of the patients; nephrotoxicity was the most common side effect.

## Discussion

Recently, combinations of steroidal and steroid-sparing immunosuppressive therapies for VKH were proposed to prevent chronic recurrent disease (6). In the present study, we focused on the clinical characteristics and treatment efficacies of MTX and CsA in patients with VKH, as well as the use of ADA and low-dose MTX combination therapy for the treatment of late-stage VKH in Japanese patients.

In this study, 54.8% of all patients with VKH were treated with steroid-sparing immunosuppressive therapies, including MTX, CsA, and ADA. In many instances, CsA was chosen first, as this is approved for the treatment of non-infectious uveitis. Patients with early-stage VKH received immunosuppressive therapy approximately within 1 month of diagnosis, during the tapering of oral corticosteroids. Anterior chamber cells and exudative retinal detachment were more prominent in patients with early-stage VKH, whereas choroidal thickening/folding was present in patients with early- and late-stage VKH at baseline when they received immunosuppressive therapy. Sunset glow fundus, a characteristic depigmentation sign of VKH, was present in all patients with late-stage VKH. Neurological and cutaneous features were slightly less prominent in patients who received immunosuppressive therapy at the early-stage of disease, which may have been improved because of the aggressive corticosteroid therapy. Inflammatory relapse occurred in 50.0% and 22.7% of patients treated with MTX or CsA, respectively. Notably, the rate of side effects requiring discontinuation of medication was higher in patients treated with CsA than in patients treated with MTX. In Japan, the approved maximum dose of MTX is 16 mg/week because of the increased prevalence of adverse events in Japanese patients (13). The higher recurrence rate in the MTX group may be associated with the insufficient MTX dose, which had been adjusted because of side effects. Furthermore, regardless of the concurrent initiation of MTX with systemic corticosteroid treatment, its effects were not apparent until a few months after administration, unlike immediate-acting agents such as CsA and ADA (14). Thus, the use of delayed-acting agents such as MTX alone may be inappropriate to prevent ongoing subclinical choroidal inflammation in patients with early-stage VKH.

As we previously suggested, VKH may require stronger anti-inflammatory treatment, compared with other uveitis etiologies (15). Adalimumab is more effective when used in combination with disease-modifying antirheumatic drugs (e.g., MTX) than when used alone (16–18). In the current study, 13 out of 14 patients who were treated with ADA received the treatment at late-stage. Of these 13 patients, relapse occurred in 11, which required ADA and MTX combination therapy. After the onset of late-stage VKH, the immunosuppressive effect of ADA alone might unfortunately be insufficient to control ocular flares and to prevent further functional and morphological dysfunctions. In this study, ADA and MTX combination therapy was successful in 72.7% of patients with late-stage VKH; the median recurrence-free period was 20 months. Previously, an adequate concentration of MTX to suppress inflammation with limited hepatotoxicity in Japanese patients with RA was achieved using a dose of 10–12 mg/week (19). Previously we reported that the median maximum dose of MTX that achieved inflammatory quiescence in patients with sclerouveitis was 16 mg/week, and the inflammatory control of non-infectious uveitis was achieved with the median dose of 12 mg/week (20, 21). In this study, the median concomitant MTX dose required at inflammatory quiescence without any side effects was 8.0 mg/week (0.13 mg/kg), which is considered low-dose therapy. This suggests that combination therapy involving ADA and MTX (at a dose lower than generally considered adequate for monotherapy) may be sufficient to achieve inflammatory control in patients in late-stage VKH. In our study, 81.8% of patients who were treated with the combination therapy required MTX within 6 months after ADA administration. Because of the slow onset of action of MTX, we presume that it is preferable to initiate MTX concomitantly with ADA in patients with late-stage VKH. To the best of our knowledge, this study includes the largest series of patients with VKH who were treated with ADA and MTX combination therapy.

According to a previous study conducted in Japan, ~61% of patients receiving systemic corticosteroid monotherapy eventually developed sunset glow fundus (5, 14, 22–25). Thus, if the goal of successful treatment is preventing the onset of late-stage disease, there is a risk of over-treatment in one-third of patients if steroid-sparing immunosuppressive therapy is administered at the initiation of systemic corticosteroid therapy. However, when the consequences of late-stage disease (e.g., cataract, glaucoma, contrast sensitivity, color vision deficits, and extraocular complications) are weighed against the potential side effects of steroid-sparing immunosuppressive therapy, early ADA or ADA and MTX combination may be appropriate, especially in younger patients (14).

There were several limitations in this study. First, the number of patients was limited; thus, a multicenter study in Japan is needed to determine the efficacy and the rate of side effects associated with MTX and CsA, as well as ADA and MTX combination therapy, among patients with late-stage VKH in a broader population. A direct comparison of ADA monotherapy, and ADA and MTX combination therapy should also be conducted. Second, a longer follow-up period is needed because of the chronic nature of VKH. Further assessment of ADA and MTX combination therapy in patients with early- and late-stage VKH, as well as the possibility of the suspension of therapy should be conducted. Third, the duration from VKH onset to first steroid-sparing immunosuppressive therapy varied among patients in this study. A better analysis of the treatment results of MTX and CsA might have been possible if all patients had received treatment with consistent timing. Fourth, the measurement of ADA trough levels and anti-ADA antibody levels would be useful to assess their relationship with treatment suspension. The presence of antinuclear antibodies may be a predictable consequence of sufficient anti-tumor necrosis factor-alpha blockade, which may aid the treatment suspension (26, 27). Last, this was a retrospective study and thus included various patients with different treatment backgrounds. A prospective study is needed to investigate the use of ADA and MTX therapy in patients with VKH.

In conclusion, this study demonstrated the clinical characteristics of VKH in Japanese patients; a high relapse rate was observed in patients treated with MTX and a high rate of side effects requiring discontinuation was observed in patients treated with CsA. This study showed that patients with late-stage VKH may achieve better disease control with ADA and low-dose MTX combination therapy.

## Data Availability Statement

The data that support the findings of this study are available on request from the corresponding author, Yoshiaki Kiuchi. The data are not publicly available due to their containing information that could compromise the privacy of research participants.

## Ethics Statement

The studies involving human participants were reviewed and approved by University of Hiroshima Institutional Review Board. The patients/participants provided their written informed consent to participate in this study. Written informed consent was obtained from the individual(s) for the publication of any potentially identifiable data included in this article.

## Author Contributions

TH and YH contributed to the conception, design of the study, and organized the database. TH performed the statistical analyses and wrote the first draft of the manuscript. TH, YH, and YK contributed to the manuscript revision and reading, and approved the submitted version. All authors contributed to the article and approved the submitted version.

## Conflict of Interest

The authors declare that the research was conducted in the absence of any commercial or financial relationships that could be construed as a potential conflict of interest.

## Publisher's Note

All claims expressed in this article are solely those of the authors and do not necessarily represent those of their affiliated organizations, or those of the publisher, the editors and the reviewers. Any product that may be evaluated in this article, or claim that may be made by its manufacturer, is not guaranteed or endorsed by the publisher.
